# Relationship Between Food Addiction, Night Eating Syndrome and Depression Level in University Students and Affecting Factors: A Cross‐Sectional Study in Türkiye

**DOI:** 10.1002/fsn3.4654

**Published:** 2025-01-31

**Authors:** Sinem Erem, Deniz Eren

**Affiliations:** ^1^ Department of Nutrition and Dietetics, Faculty of Health Sciences Trakya University Edirne Türkiye; ^2^ Department of Nursing, Faculty of Health Sciences Trakya University Edirne Türkiye

**Keywords:** depression, food addiction, night‐eating syndrome, university students

## Abstract

Food addiction, night eating, and depression levels alone have been associated with obesity and various chronic diseases in university students, but the relationship of these factors has been rarely mentioned in the literature. This research was conducted to determine the food addiction, night eating, and depression status of university students. The study consisted of 1009 (84.2% female, mean BMI 21.93 ± 3.42 kg/m^2^, 73.0% normal body weight) university students. Research data were collected using a web‐based questionnaire, which included a personal information form about students' sociodemographic characteristics, nutritional habits and anthropometric measurements (body weight, height), the Yale Food Addiction Scale (YFAS), the Night Eating Questionnaire (NEQ), and Beck Depression Inventory (BDI). Of the students, 15.2% had food addiction, 8.3% had night‐eating syndrome, and 36.2% had moderate depression. Frequency of food addiction and night‐eating syndrome were higher in those with moderate to severe depression and night‐eating syndrome was higher in those with food addiction (*p* < 0.001). A positive correlation was found between mean YFAS, NEQ, and BDI scores in the general population and both genders (*p* < 0.001). Furthermore, BMI levels, having received psychological treatment, waking up and eating at night, and depression level increased the likelihood of food addiction, while waking up and eating at night, and depression levels increased the likelihood of night‐eating syndrome. The findings of the study made a significant contribution to the literature in understanding food addiction, night eating, depression levels and their relationship with each other in university students. Multi‐center studies in which the effects of these three factors are examined together should be conducted on different student populations. Students should be given training on healthy nutrition and mental health protection, and their awareness should be raised.

## Graphical abstract

Food addiction, night eating syndrome, and depression are the three factors affecting university students. There was a positive correlation between mean YFAS, NEQ, and BDI scores in the general population and both genders. BMI levels, having received psychological treatment, waking up and eating at night, and depression level increased the likelihood of food addiction. Meanwhile, waking up and eating at night and BMI levels increased the likelihood of night‐eating syndrome.

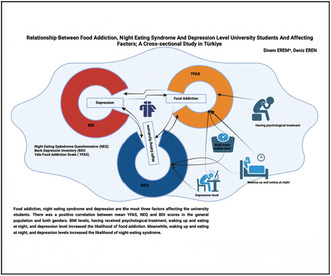

## 1 Introduction

During university education, individuals mature physically, spiritually, and socially and begin to develop adult‐specific behaviors. This period is referred to as “young adulthood” or “emerging adulthood,” which refers to the transition to adulthood (Hochberg and Konner 2020; Wider et al. 2023). Youth has been described as the period of life between 15 and 29 years by the Organization for Economic Co‐operation and Development (OECD) and between 15 and 24 years by the International Labor Organization (ILO), the United Nations Educational, Scientific and Cultural Organization (UNESCO), the World Bank (World Bank), the United Nations (UN), and our country (OECD 2021). With the transition to university, many individuals prepare meals for the first time without their parents and form their own eating habits by undertaking food shopping (Kabir, Miah, and Islam 2018). Especially during this period, failure to adapt to academic life can lead to stress, alcohol and cigarette use, skipping meals, turning to fast food, eating disorders (binge eating, night‐eating syndrome, anorexia nervosa, and bulimia nervosa), obesity, and depression (Brenda Echeverri et al. 2023; Choi 2020; Ribeiro et al. 2023; Ruddock et al. 2017; Vila‐Martí, Elío, and Sumalla‐Cano 2021).

Food addiction (FA) refers to addictive behavior associated with certain types of food, especially those that are palatable and high in energy. Criteria for FA defined by the Diagnostic and Statistical Manual of Mental Disorders (DSM‐5) have been defined as the continuation of highly processed food intake despite negative physical, mental, and social consequences, the development of tolerance, and persistent unsuccessful attempts to reduce food intake. The Yale Food Addiction Scale (YFAS) is the most frequently used measurement tool by researchers to assess FA and helps do an evaluation by taking into account the Diagnostic and Statistical Manual of Mental Disorders (DSM) criteria (Cristina Romero‐Blanco et al. 2021; Gordon et al. 2018; Hurel et al. 2021). Hauck et al. stated that FA was more common in young individuals 18–29 years old (Hauck et al. 2017). The prevalence of FA ranges from 5.4% in the general healthy population to 50% in individuals diagnosed with binge eating, 53.7% in patients after bariatric surgery, and 54.5% in electronic sports players (Arslan et al. 2024; Cheah and Chin 2024; Horsager et al. 2023; Praxedes et al. 2022; Vasiliu 2022). Additionally, Nolan and Geliebter emphasized that FA was associated with night eating, overweight or obesity, depression, emotional eating, and sleep problems (Nolan and Geliebter 2019).

Night‐eating syndrome (NES) is generally classified under eating disorders, and recently, for the first time, it has been included under the heading “other specified feeding and eating disorders” in the Diagnostic and Statistical Manual of Mental Disorders (DSM‐5). Night‐eating syndrome is an eating disorder characterized by a delayed circadian pattern of food intake. For a person to be diagnosed with NES under this heading, at least three of the following five criteria must be present: (a) a lack of desire to eat or skipping breakfast four or more times a week; (b) having a strong urge to eat between dinner and bedtime and/or throughout the night; (c) difficulty falling asleep and/or maintaining sleep at least four nights a week; (d) believing that you need to eat to sleep, and (e) being in a depressed or worse mood in the evening (Lavery and Frum‐Vassallo 2022). It has been stated that NES is 6–10 times more common in individuals with BMI ≥ 30 kg/m^2^ than in the general population (Sayed Ahmed et al. 2024). In a study conducted in Malaysia, it was found that 15% of university students showed symptoms of NES (Gan, Chin, and Law 2019). In addition, studies conducted in recent years indicated that the frequency of NES was 1.5%–4.6% in the general population and 15% especially in the presence of depression, 49.36% of medical students had NES and 27.84% had depression, students who were diagnosed with NES experienced depression statistically significantly compared to those who were not, and that FA was between 25% and 29% in sample groups clinically diagnosed with depression (Haneef and Almuammar 2024; Mills et al. 2020; Zafar and Zaidi 2020). Research shows that university students have higher levels of depression than other populations.

Considering that half of all mental disorders occur by the age of 14 and 62.5%–75% by the age of 24, university students appear to be at high risk for mental health problems (Roldán‐Espínola et al. 2024). The prevalence of depression also varies according to socio‐demographic factors, such as gender, age, environment, and personal character, income level, and health‐related behaviors (Alhemedi et al. 2023). Elkholy et al. claimed that major depressive disorders, FA, NES, and obesity were directly related (Elkholy, Molokhia, and Rizk 2023). Goncalves et al. (2022) stated that a negative emotional state increased FA and night‐eating behaviors in university students.

Therefore, addressing nutritional disorders and related mental health problems seen in young university students will be useful, especially in terms of effectively introducing the protective measures that need to be taken. Reflecting on this information, this study aimed to determine FA, NES, and depression among university students aged 18–30.

## 2 Methods

This cross‐sectional study was conducted between March and June 2023. The sample of the research consisted of 1.009 university students aged 18–30, studying at Trakya University Faculty of Health Sciences and volunteering to participate in the study. Research data were collected online using a questionnaire created on Google Forms. The link to the online questionnaire was sent to the students' phones at the end of the lesson in the relevant classes using an online messaging tool (WhatsApp, Messenger, etc.). Students studying at Trakya University Faculty of Health Sciences were included in the research. The research population consisted of 2550 students (752, Nursing; 444, Physiotherapy and Rehabilitation; 399, Nutrition and Dietetics; 687, Health Management; and 268, Audiology). The sample size was estimated using the sampling of the known population formula (rate of incidence: 50.0%), and it was determined that a minimum of 334 (98, Nursing; 59, Physiotherapy and Rehabilitation; 52, Nutrition and Dietetics; 90, Health Management; and 35, Audiology) students were needed. A stratified random sampling method was used to determine the estimated number of students to be recruited from each department (Sumbuloglu and Sumbuloglu 2009). The research was completed with a total of 1009 students, including 240 from the Department of Nursing, 170 from the Department of Physiotherapy and Rehabilitation, 346 from the Department of Nutrition and Dietetics, 112 from the Department of Health Management, and 141 from the Department of Audiology. At the outset, the approval of the Trakya University Faculty of Medicine Scientific Research Ethics Committee (number: 2023/44—date: 13.02.2023) and the permission of the Trakya University Faculty of Health Sciences (number: 422522—date: 15.03.2023) were obtained. The study was conducted in accordance with the principles of the Declaration of Helsinki.

The questionnaire included questions about participants' descriptive characteristics (age, gender, marital status, etc.), eating habits (meal patterns, appetite), food addictions (YFAS), night eating syndrome (NEQ), depression status (Beck Depression Inventory (BDI)), and anthropometric measurements (body weight, height).

### 2.1 The Yale Food Addiction Scale (YFAS)

This scale was developed by Gearhardt, White, and Potenza (2011) and Cronbach's alpha value was found to be 0.86 in the original validity and reliability study of the scale. The scale was first adapted to Turkish by Bayraktar, Erkman, and Kurtulus (2012) and a validity and reliability study was conducted (Bayraktar, Erkman, and Kurtulus 2012). Cronbach's alpha value of the scale was found to be 0.93. It consists of 27 items and is used to measure individuals' addiction‐like eating behaviors for the types of food they have consumed in the last 12 months. The scale was created by adapting the substance addiction criteria in DSM‐IV to food addiction (Burmeister et al. 2013).

The first 16 questions on the scale are five‐point Likert type and are evaluated between 0 and 4 points (0—never, 1—once a month, 3—two or three times a week, 4—four or more times a week). Questions 17–24 are yes/no type and are scored between 0 and 1 point. The 25th item questions how many times the individual has tried to reduce or stop eating certain foods in the past year. Questions 17, 18, and 23 are not included in the scoring because they are precursors to other questions. For a food addiction diagnosis, in addition to meeting at least three of the seven diagnostic criteria (3 points and above), clinical significance (1 point) is required. A score of 1 from one of the 15th and 16th questions shows clinical significance. The scale consists of a total of 8 symptoms. These are taking the substance for a long time and more than planned (Symptom 1); intense desire to quit or repeated unsuccessful attempts to quit (Symptom 2); spending too much time obtaining, using, and quitting (Symptom 3); giving up or reducing social activities (Symptom 4); continuing to use the substance despite knowing negative consequences (Symptom 5); showing tolerance (Symptom 6); using the substance to reduce withdrawal symptoms (Symptom 7); clinically significant problems due to the use of the substance (Symptom 8).

Food addiction criterion scores are calculated separately for each symptom. If the total score of the questions for each symptom is ≥ 1, the criterion score is evaluated as 1, and if it is zero, the criterion score is evaluated as zero. The first 16 questions are scored as follows: never = 0, once a month = 1, twice to four times a month = 2, twice a week = 3, and more than four times a week = 4. Questions are scored as follows: 0, 1, 2, or 3 responses to questions 1, 2, 4, 6, and 25 are evaluated as 0 points and responding 4 is evaluated as 1 point; 0, 1, or 2 responses to questions 3, 5, 7, 9, 12, 13, 14, 15, and 16 are evaluated as 0 points and responses 3 and 4 are evaluated as 1 point; 0 and 1 responses to questions 8, 10, and 11 are evaluated as 0 points and responses 2, 3, and 4 are evaluated as 1 point; “no” response to questions 19, 20, 21, and 22 are evaluated as zero points and a “yes” answer to these questions is evaluated as 1 point; “no” response to question 24 is evaluated as 1 point and a “yes” response is evaluated as 0 points. Questions 17, 18, and 23 are not included in the scoring (Sevincer et al. 2014).

### 2.2 The Night Eating Questionnaire (NEQ)

This scale was developed and its validity and reliability study was performed by Allison et al. in 2008. Cronbach alpha value in the original validity study was found to be 0.70. It consists of 14 questions. As a result of the Turkish validity and reliability conducted by Atasoy et al. (2014), the alpha coefficient was found to be 0.69 (Atasoy et al. 2014). The first nine questions are answered by all individuals. Questions 10 and 12 are for individuals who wake up at night, and 13 and 14 are for those who have night eating habits. Except for the 7th item, items on the questionnaire are evaluated using a five‐point Likert‐type scale between 0 and 4 points. The seventh item is evaluated as 0 points in those who have no mood changes during the day. Items 1, 4, and 14 are reverse‐scored. Item 13 is not included in the scoring (Allison et al. 2008). The total scale score varies between 0 and 52.

In the original validity and reliability study of the scale, the positive predictive value was reported as 40.7% for a score of ≥ 25 and 72.7% for a score of ≥ 30. In the same study, the negative predictive value for NEQ was high at both cut‐off points of 25 and 30 points (95.2% and 94%, respectively).

### 2.3 Beck Depression Inventory (BDI)

This is a 21‐item, four‐choice inventory developed by Beck et al. (1961) and its Cronbach alpha value was calculated as 0.88 (Beck et al. 1961). Items are evaluated between 0 and 3 points depending on the level of depression (0: positive statements about depression and 3: negative statements about depression). The score range is 0 to 63. As the total score increases, the level of depression increases, as well. Total scores are interpreted as follows: 0–9, “minimal depression”; 10–16, “mild depression”; 17–29, “moderate depression”; 30–63, “severe depression.” The validity and reliability study of the Turkish adaptation of the scale was conducted by Hisli (1989), and Cronbach's alpha value was found to be 0.80. In this study, students' depression levels were examined in two groups: minimal‐mild (16 points and below) and moderate–severe (17 points and above) depression.

### 2.4 Evaluation of Anthropometric Measurements

Individuals' body weight (last three months) and height were recorded based on self‐report. BMI values were calculated using students' body weight and height. BMI percentile values for individuals aged ≤ 19 were evaluated as follows: < 5, malnutrition; ≥ 5–< 15, underweight; ≥ 15–< 85, normal weight; ≥ 85–< 95, slightly overweight. For individuals aged 20 and over, BMI values were as follows: < 18.5 kg/m2, underweight; 18.5–24.9 kg/m2, normal weight; 25.0–29.9 kg/m2, slightly overweight; 30.0–34.9 kg/m2, first degree obese (WHO 2010; Pekcan, 2008).

#### 2.4.1 Statistical Analysis

The data obtained in the study were analyzed on the SPSS (Statistical Package for Social Sciences) for Windows 25.0 and AMOS 21 software. Descriptive statistical methods (frequency, percentage, mean, and standard deviation values) were utilized for evaluating the data. Reliability analysis is carried out to test whether the statements on a scale are consistent with each other (Ural and Kilic 2006). For the tests and results to be reliable, the measurements must be reliable. In this context, the reliability of the scale was examined with Cronbach's alpha coefficient. In addition to normality tests to determine the normality distribution of the data, histogram, Q‐Q plot and box‐plot, skewness and kurtosis, and variation coefficients were employed. Chi‐square test was employed to evaluate the relationship between independent categorical variables, and logistic regression analysis to explain the dependent categorical variables (Büyüköztürk 2011; Hayran and Hayran 2011). Other methods used in the analyses included Spearman correlation analysis to determine the relationship between measurement tools. The correlation coefficient was interpreted as follows: 0.00–0.10, no relationship; 0.10–0.39, weak relationship; 0.40–0.69, moderate relationship; 0.70–0.89, strong relationship; and 0.90–1.00 as a very strong relationship (Schober, Boer, and Schwarte 2018). Reliability was accepted as 95.0% in all statistical analyses.

## 3 Results

The distribution of students' FA, night‐eating syndrome, and depression levels according to their sociodemographic characteristics is given in Table 1. The mean age of the students was 21.04 ± 1.65 years. The mean BMI was 21.93 ± 3.42 kg/m^2^, and 74.3% of the students had normal body weight. In addition, 34.3% of them were from the nutrition and dietetics department, 84.2% were female, and 98.9% were single. It was also determined that 49.2% of the students were staying in state dormitories, 18.1% had received psychological treatment/therapy, 28.5% smoked, and 34.5% used alcohol. When the eating habits of the students were examined, it was found that 80.5% skipped main meals, 95.3% skipped snacks, and 11.3% woke up and ate at night. It was determined that 31.4% of the students with FA were from the nutrition and dietetics department and that 30.1% were from the nursing department. Of the students with FA, 30.7% had received psychological treatment/therapy, 36.6% smoked, 82.4% skipped main meals, 91.5% skipped snacks, 19.0% woke up at night and ate, and 67.3% had a normal body weight. Of the students with NES, 22.6% studied in the nutrition and dietetics department, 26.2% studied in the nursing department, 28.6% had received psychological treatment/therapy, 39.3% smoked, 88.1% skipped main meals, 90.5% skipped snacks, 32.1% woke up and ate at night, and 57.1% had a normal body weight. Of the students with moderate to severe depression levels, 31.8% were from the nutrition and dietetics department, 18.6% were from the nursing department, 22.2% had received psychological treatment/therapy, 32.9% smoked, 84.9% skipped meals, 94.2% skipped snacks, and 14.5% woke up and ate at night (Table 1).

**TABLE 1:** Distribution of food addiction, night‐eating syndrome, and depression levels according to sociodemographic characteristics.

Factors	Total *n* (%)	FA		NES		Depression level	
		No *n* (%)	Yes *n* (%)	No *n* (%)	Yes *n* (%)	Minimal‐mild *n* (%)	Moderate–severe *n* (%)
Gender							
Female	850 (84.2)	715 (83.5)	135 (88.2)	776 (83.9)	74 (88.1)	537 (83.4)	313 (85.8)
Male	159 (15.8)	141 (16.5)	18 (11.8)	149 (16.1)	10 (11.9)	107 (16.6)	52 (14.2)
Department							
Nutrition and dietetics	346 (34.3)	298 (34.8)	48 (31.4)	327 (35.4)	19 (22.6)	230 (35.7)	116 (31.8)
PTR*	170 (16.8)	140 (16.4)	30 (19.6)	157 (17)	13 (15.5)	107 (16.6)	63 (17.3)
Nursing	240 (23.8)	194 (22.7)	46 (30.1)	218 (23.6)	22 (26.2)	172 (26.7)	68 (18.6)
Audiology	141 (14.0)	119 (13.9)	22 (14.4)	129 (13.9)	12 (14.3)	74 (11.5)	67 (18.4)
Healthcare management	112 (11.1)	105 (12.3)	7 (4.6)	94 (10.2)	18 (21.4)	74 (11.5)	67 (18.4)
Marital status							
Married	998 (98.9)	847 (98.9)	151 (98.7)	915 (98.9)	83 (98.8)	636 (98.8)	362 (99.2)
Single	11 (1.1)	9 (1.1)	2 (1.3)	10 (1.1)	1 (1.2)	8 (1.2)	3 (0.8)
Place of residence							
Family home	153 (15.2)	137 (16.0)	16 (10.5)	145 (15.7)	8 (9.5)	93 (14.4)	60 (16.4)
State dormitory	496 (49.2)	419 (48.9)	77 (50.3)	444 (48.0)	52 (61.9)	311 (48.3)	185 (50.7)
Private dormitory	117 (11.6)	97 (11.3)	20 (13.1)	110 (11.9)	7 (8.3)	77 (12.0)	40 (11.0)
With friends	132 (13.1)	105 (12.3)	27 (17.6)	123 (13.3)	9 (10.7)	86 (13.4)	46 (12.6)
Home alone	111 (11.0)	98 (11.4)	13 (8.5)	103 (11.1)	8 (9.5)	77 (12.0)	34 (9.3)
Status of receiving psychological treatment/therapy							
Yes	183 (18.1)	136 (15.9)	47 (30.7)	159 (17.2)	24 (28.6)	102 (15.8)	81 (22.2)
No	826 (81.9)	720 (84.1)	106 (69.3)	766 (82.8)	60 (71.4)	542 (84.2)	284 (77.8)
Smoking							
Yes	288 (28.9)	232 (27.1)	56 (36.6)	255 (27.6)	33 (39.3)	168 (26.1)	120 (32.9)
No	721 (81.9)	624 (72.9)	97 (63.4)	670 (72.4)	51 (60.7)	476 (73.9)	245 (67.1)
Alcohol consumption							
Yes	348 (34.5)	290 (33.9)	58 (37.9)	311 (33.6)	37 (44.0)	211 (32.8)	137 (37.5)
No	661 (65.5)	566 (66.1)	95 (62.1)	614 (66.4)	47 (56.0)	433 (67.2)	228 (62.5)
Skipping main meals							
Yes	812 (80.5)	686 (80.1)	126 (82.4)	738 (79.8)	74 (88.1)	502 (78)	310 (84.9)
No	197 (19.5)	170 (19.9)	27 (17.6)	187 (20.2)	10 (11.9)	142 (22)	55 (15.1)
Skipping snacks							
Yes	962 (95.3)	822 (96.0)	140 (91.5)	886 (95.8)	76 (90.5)	618 (96.0)	344 (94.2)
No	47 (4.7)	34 (4.0)	13 (8.5)	39 (4.2)	8 (9.5)	26 (4.0)	21 (5.8)
Waking up and eating at night							
Yes	114 (11.3)	85 (9.9)	29 (19.0)	87 (9.4)	27 (32.1)	61 (9.5)	53 (14.5)
No	895 (88.7)	771 (90.1)	124 (81.0)	838 (90.6)	57 (67.9)	583 (90.5)	312 (85.5)
BMI classification (kg/m^2^)							
Underweight	117 (11.6)	101 (11.8)	16 (10.5)	106 (11.5)	11 (13.1)	66 (10.2)	51 (14.0)
Normal	739 (73.2)	636 (74.3)	103 (67.3)	691 (74.7)	48 (57.1)	479 (74.4)	260 (71.2)
Overweight	137 (13.6)	112 (13.1)	25 (16.3)	116 (12.5)	21 (25.0)	92 (14.3)	45 (12.3)
Obese	16 (1.6)	7 (0.8)	9 (5.9)	12 (1.3)	4 (4.8)	7 (1.1)	9 (2.5)

Abbreviations: BMI, body mass index; PTR, physiotherapy and rehabilitation.

**FIGURE 1:**
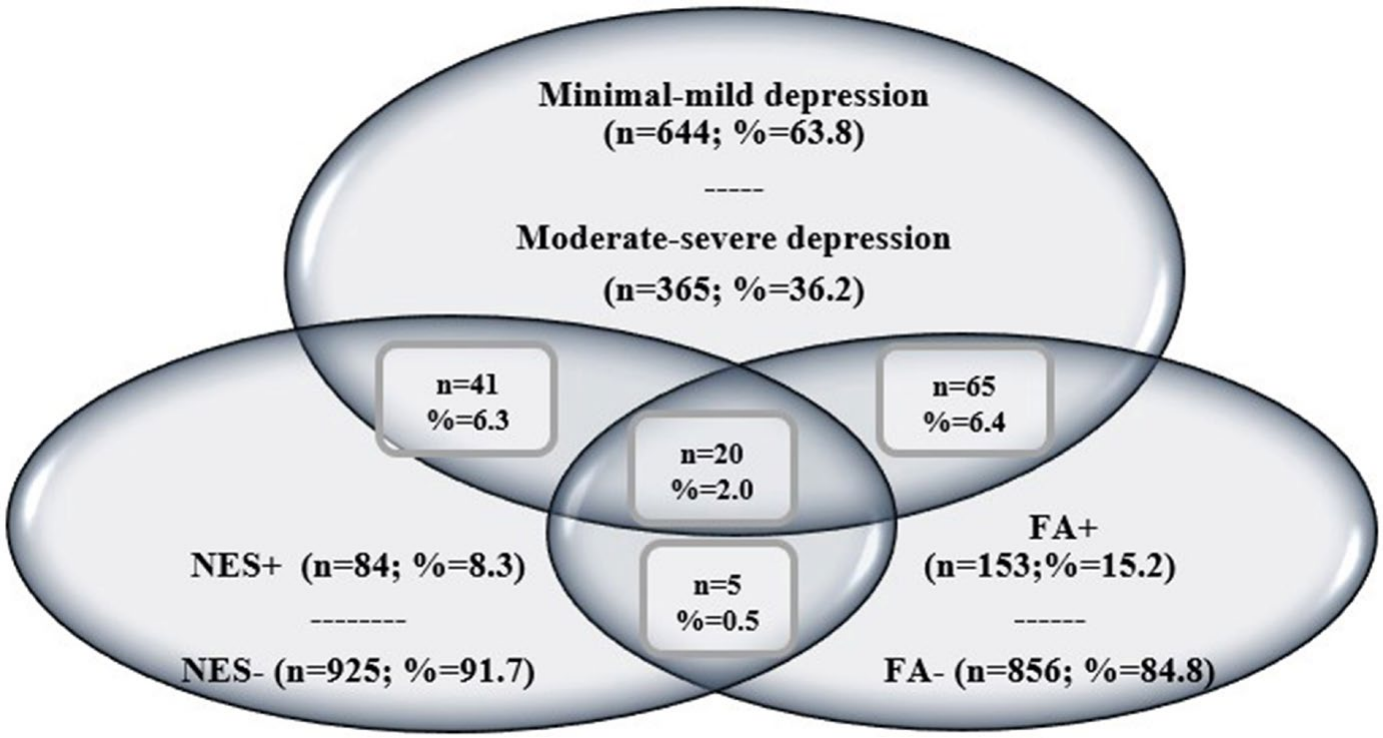
Distribution of students' food addiction, night‐eating syndrome, and depression status. FA‐, No Food Addiction; FA+, Having Food Addiction; NES‐, No Night Eating Syndrome; NES+, Having Night Eating Syndrome.

The distributions of FA, NES, and depression levels of the students participating in the study are shown in Figure 1. Of the students, 15.2% had food addiction, and 8.3% had night eating behavior. Additionally, the depression levels were minimal‐mild in 63.8% and moderate–severe in 36.2%. Two percent of the general sample had FA, moderate to severe depression, and night eating habits. The mean scores of the students were 3.11 ± 1.79 for FA, 16.45 ± 5.61 for NES, and 14.33 ± 9.90 for BDI (Table 2).

**TABLE 2:** Descriptive characteristics of the YFAS, NEQ, and BDI scales.

Scales		*n*(%)	$\bar{X}$ ± SD (Min.‐Max.)
**YFAS**	FA—	856 (84.8)	3.11 ± 1.79 (0–8)
	FA+	153 (15.2)	
**NEQ**	NES—	925 (91.7)	16.45 ± 5.61 (4–41)
	NES+	84 (8.3)	
**BDI**	Minimal‐mild depression	644 (63.8)	14.33 ± 9.90 (0–63)
	Moderate–severe depression	365 (36.2)	

Abbreviations: $\bar{X}$, mean; BDI, beck depression inventory; FA−, no food addiction; FA+, have a food addiction; NEQ, night eating questionnaire; NES−, no night eating syndrome; NES+, have a night eating syndrome; SD, standard deviation; YFAS, yale food addiction scale.

A statistically significant relationship was found between students' FA and NES and their depression levels (*p* < 0.05). Additionally, there was a statistically significant relationship (*p* < 0.05) between night‐eating syndrome and food addiction among the students participating in the study. It was found that 23.3% of the students with moderate to severe depression and 10.6% of those with minimal to mild depression had food addiction. Also, 16.7% of the students with moderate to severe depression and 3.6% of those with minimal to mild depression had a habit of eating at night. Moreover, 16.3% of the students with FA had NES (Table 3).

**TABLE 3:** Relationship between depression level, FA, and NES status.

Scales		Depression level				*x* ^2^	*p*	Phi coefficient (φ)
		Minimal‐mild		Moderate–severe				
		***n***	**%**	***n***	**%**			
**FA**	FA‐	576	89.4	280	76.7	29.341	0.000*	0.171
	FA+	68	10.6	85	23.3			
**NES**	NES—	621	96.4	304	83.3	52.711	0.000*	0.229
	NES+	23	3.6	61	16.7			

		**FA**						
		**No**		**Yes**				
		***n***	%	***n***	**%**			
**NES**	NES—	797	79.0	128	83.7	15.179	0.000*	0.123
	NES+	59	5.8	25	16.3			

* *p* < 0.05; Chi‐square test.

Abbreviations: FA, food addiction; NES−, no night eating syndrome; NES+, have a night eating syndrome.

**FIGURE 2:**
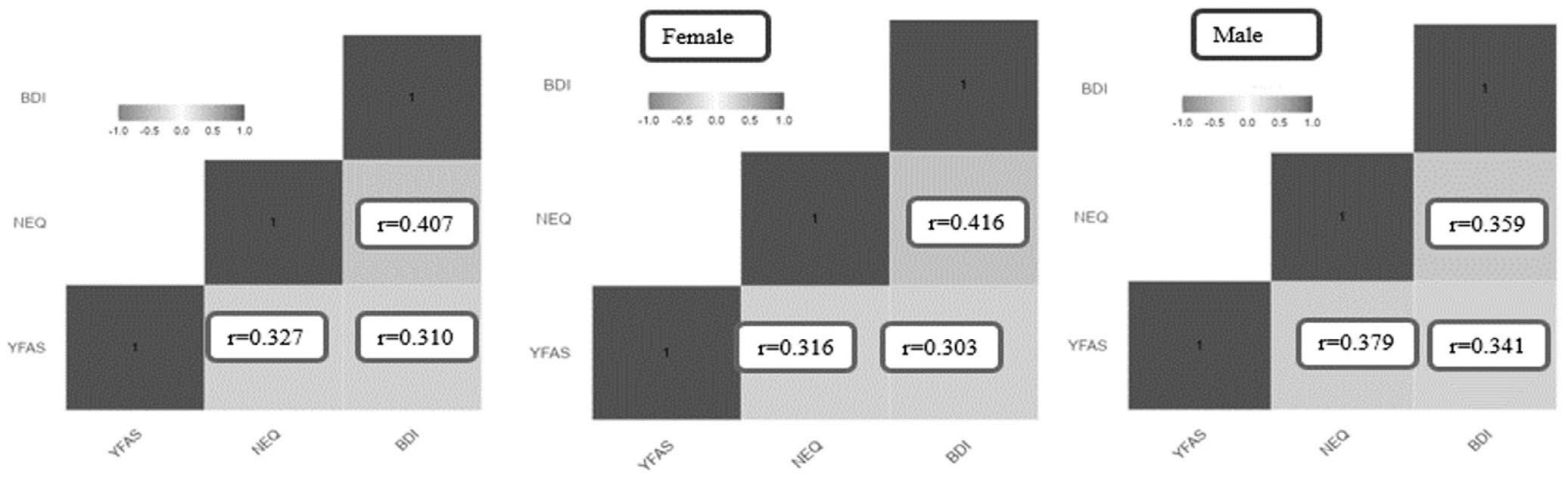
Relationships between mean YFAS, NEQ, and BDI scores according to gender. Spearman correlation coefficient (r), *p*‐<‐0.001. BDI, Beck Depression Inventory; NEQ, Night Eating Questionnaire; YFAS, Yale Food Addiction Scale.

There was a significant, positive, and weak relationship between students' mean YFAS and NEQ scores (*r* = 0.327, *p* = 0.000) and between NEQ and BDI scores (*r* = 0.310, *p* = 0.000) and a significant, positive, and moderate relationship between mean YFAS and BDI scores (*r* = 0.407 *p* = 0.000). The relationship between female students' mean YFAS and NEQ scores (*r* = 0.303 *p* = 0.000), NEQ and BDI (*r* = 0.316 *p* = 0.000) scores, and male students' mean YFAS and NEQ scores (*r* = 0.379 *p* = 0.000), YFAS and BDI scores (*r* = 0.359 *p* = 0.000), and NEQ and BDI scores (*r* = 0.341 *p* = 0.000) was significant, positive, and weak. Additionally, the relationship between female students' mean YFAS and BDI scores was significant, positive, and moderate (*r* = 0.416 *p* = 0.000) (Figure 2).

When the factors affecting students' FA and NES were examined with logistic regression analysis, it was seen that there was a model fit (Hosmer‐Lemeshow test *p*‐value = 0.639; 0.922, respectively). In the model established in the study, four independent variables were found to be statistically significant, and the explanatory power of the model was determined to be 12.1% (Nagelkerke R^2^ = 0.121). According to the results of the model, it was determined that as students' BMI value increased, the probability of having FA increased, as well (Exp(B) = 1.110). Food addiction was found to be higher in students who had received psychological treatment/therapy than those who had not (Exp(B) = 2.125), those who had night‐eating syndrome than those who did not (Exp(B) = 1.805), and those who had moderate to severe depression than those with minimal‐mild depression (Exp(B) = 2.344) (Table 4). Three independent variables were found to be statistically significant in the model established for night‐eating syndrome, and the explanatory power of the model was found to be 20.7% (Nagelkerke R^2^ = 0.207). According to the results of the model, as the BMI value of the students increased, the probability of having night‐eating syndrome increased, as well (Exp(B) = 1.110). Night‐eating syndrome was more common in students who woke up at night and ate than those who did not have night eating habits (Exp(B) = 4.073). Students with moderate–severe depression had higher levels of night‐eating syndrome than those with minimal‐mild depression (Exp(B) = 5.069) (Table 4).

**TABLE 4:** Factors affecting students' FA and NES.

		FA						NES				
Factors	OR	CI (%95)		*β*	SE	*p*	OR	CI (%95)		*β*	SE	*p*
		Lower	Upper					Lower	Upper			
Age	0.908	0.807	1.021	−0.096	0.060	0.108	0.994	0.857	1.154	−0.006	0.076	0.939
BMI(kg/m^2^)	1.110	1.055	1.168	0.104	0.026	0.000*	1.110	1.044	1.179	0.104	0.031	0.001*
Department	0.962	0.650	1.424	−0.038	0.200	0.847	0.646	0.367	1.137	−0.437	0.289	0.130
Status of receiving therapy	2.125	1.402	3.222	0.754	0.212	0.000*	1.571	0.892	2.770	0.452	0.289	0.118
Smoking	0.828	0.560	1.225	−0.189	0.200	0.345	0.886	0.529	1.484	−0.121	0.263	0.646
Skipping snacks	1.796	0.850	3.795	0.585	0.382	0.125	1.296	0.498	3.374	0.259	0.488	0.595
Waking up at night and eating	1.805	1.100	2.961	0.590	0.253	0.019*	4.073	2.334	7.109	1.404	0.284	0.000*
Depression	2.344	1.633	3.364	0.852	0.184	0.000*	5.069	3.023	8.498	1.623	0.264	0.000*
Constant	0.078			−2.548	1.377	0.064	0.004			−5.649	1.757	0.001*
Nagelkerke *R* ^2^ = 0.121; Hosmer‐Lemeshow =6.071;*p* = 0.639							Nagelkerke *R* ^2^ = 0.207; Hosmer‐Lemeshow =3.1944;*p* = 0.922					

* *p* < 0.05.

Abbreviations: CI, confidence interval; FA, food addiction; NES, night eating syndrome; OR, odds ratio; SE, standard error.

## 4 Discussion

This research was conducted to determine the levels of FA, NES, and depression in university students. Food addiction was detected in 15.2% of the students participating in the research (Table 2 and Figure 1). When studies conducted on university students were examined, the prevalence of FA was 18.6% in the study by Reivan Ortiz et al. (2024), 10.3% in the study by Sanlier, Türközü, and Toka (2016), and 17.0% in a review of nine studies conducted on individuals under the age of 35. The prevalence of NES in university students was found as 19.5% in a study conducted in Bangladesh (Khan et al. 2024), 7.3% among medical students in Saudi Arabia (Haneef and Almuammar 2024), and 17.8% in a study conducted in Greece (Blouchou et al. 2024). In this study, the prevalence of NES was found to be 8.3% (Table 2 and Figure 1). In addition to the results, while the prevalence of depression symptoms was approximately over 40.0% in the literature (Liu et al. 2023; Yichen and Chuntian 2024), in a study on the depression levels of university students in Türkiye, 72.4% had minimal‐mild depression and 27.6% had moderate to severe depression (Sanlier, Türközü, and Toka 2016). In the present study, it was found that 63.8% of the students had minimal‐mild depression, 36.2% had moderate–severe depression, 6.3% had both NES and moderate–severe depression, 6.4% had both FA and moderate–severe depression, and 0.5% had both NES and FA. Individuals meeting these three factors constituted 2.0% of the sample (Table 2 and Figure 1). These rates may indicate that depression is associated with eating disorders. Some studies conducted in the literature in recent years have also supported this issue (Al‐Shoaibi et al. 2024; Meier, Summers, and Buhlmann 2024; Soraci et al. 2024).

The eating habits of university students can be affected by factors such as the department they study in and their economic status. In our study, considering the departments where the students studied, it was determined that 31.4% of those with FA studied nutrition and dietetics and 30.1% were nursing students. When evaluated on a department basis, the highest rate of FA was found in nursing students (19.6%), which was followed by nutrition dietetics students (13.8%) (Table 1). In the literature, the rate of FA was 6.4% in a study consisting of nursing students (Romero‐Blanco et al. 2021), 7.4% in another study (Hong et al. 2020), 10.5% in a study by Unal and Ucar on nutrition and dietetics students (Unal and Ucar 2024), and 4.5% in the study by Grammatikopoulou et al. (Grammatikopoulou et al. 2018). In another study, a lower prevalence of FA was reported in nutrition and dietetics students (25.7%) than in students of other departments (27.2%) (Hong et al. 2020). In this study, although nutrition and dietetics and nursing students were at the top when looking at the general sample, a department‐based examination showed that nutrition and dietetics students had a lower rate of food addiction than the students of other departments (PTR, 17.6%; Nursing, 19.6%; Audiology, 15.6%; Health management, 6.3%). Additionally, it was determined in the study that the majority of those with night‐eating syndrome were nursing students (26.7%), and 9.2% of them had night‐eating syndrome and approximately one‐third had moderate–severe depression (Table 1). Ahmed et al. (2019) stated that night eating was more common in university students studying in departments with a heavier academic load, such as health sciences, than in students of other departments. This difference may have been caused by the fact that the departments where students studied had different curriculum weights, nutrition and dietetics students received more detailed theoretical and practical nutrition education than other departments, and that nursing students undertook intense entrepreneurial responsibilities in their internship practices and most of them were female.

Evening hyperphagia and NES in individuals cause disordered eating pathologies (Echeverri et al. 2023), especially skipping breakfast causes obesity 0.89 times more (Lee et al. 2016). While night‐eating syndrome shows a strong correlation with a diagnosis of depression in addition to obesity (Elkholy, Molokhia, and Rizk 2023), skipping breakfast is also associated with high levels of anxiety disorder (Chang et al. 2021). In a study conducted by Gluck, Geliebter, and Satov (2001) on obese individuals, it was determined that NES was likely to reduce the desire to eat during the day and slow down the weight loss process, which may cause daytime anorexia (skipping breakfast more than four days a week). In addition to these results, individuals with FA are likely to prefer fewer snacks when overeating (Ruddock and Hardman 2018), and as the number of snacks increases, NES and FA occur more frequently (Zueter and Mashal 2023). In our study, it was determined that the majority of students with FA and NES (91.5% and 90.5%, respectively) skipped snacks. Additionally, those with moderate to severe depression (78.0%) were found to skip main meals at a higher rate than those with minimal‐mild depression (78.0%) and that these students had a higher frequency of waking up and eating at night (14.5%) (Table 1). Skipping meals in university students may be due to their economic shortcomings, not wanting to spend time preparing meals, and energy imbalances between meals. Considering all these results, it becomes clear that NES, FA, and depression in students are interrelated, and also directly affect body weight change and meal skipping.

Food addiction is characterized by addiction to the consumption of palatable foods and decreased ability to reduce or stop their consumption, impulsivity, and compulsion (Jurema Santos et al. 2024; Gearhardt, White, and Potenza 2011). This situation is especially associated with the consumption of excessively processed foods in childhood and adolescence (Jurema Santos et al. 2024) and with a sedentary lifestyle during the university period, especially with elevated BMI levels (Romero‐Blanco et al. 2021). The incidence of mild obesity or obesity in individuals with FA in the literature varies between 14.7% and 26.4% in university students (Romero‐Blanco et al. 2021; Yu and Tan 2016), while this rate can reach up to 35.6% in high school students (18.0% of obese students have FA) (Meseri and Akanalci 2020). Highly processed foods rich in fat and sugar are strongly associated with FA and are consumed at high rates by individuals with FA (Schiestl et al. 2021). In our study, the rate of being slightly overweight or obese among students with FA was found to be 22.2%, and the prevalence of obesity was approximately seven times higher in those with FA (5.9%) than in those without FA (0.8%) (Table 1). This situation is caused by the fact that with the beginning of university life, students become open to external factors in terms of eating, they make food choices freely, they prefer fast food and unhealthy meals containing empty calories due to limited access to costly meals, and as the academic load increases, they have to study classes and exams at night, and they may even have to work during their university education.

Studies in the literature have shown a positive relationship between NES and increased BMI (Nolan and Geliebter 2017; Sa'ari et al. 2024; Taymur et al. 2019), and this situation has been thought to trigger obesity and metabolic syndrome (Hong et al. 2020; Saari et al. 2024). In a study conducted on Iranian adults, it was found that the risk of FA and NES increased with the choice of unhealthy diet patterns and the degree of obesity (Yousefi et al. 2024). In a study recently conducted in Türkiye on adolescents aged 11–18 years, it was found that NES and FA scores increased significantly as the degree of obesity increased (Yassibas et al. 2024). In this study, although 57.1% of the individuals with NES were found to have normal body weight, it was determined that 29.8% were overweight‐obese and approximately one‐third woke up and ate at night (Table 1). In addition, students with FA had a higher rate of NES and waking up and eating at night than those with no FA (Table 3). It was found that as the BMI level, and the frequency of waking up and eating at night increased the probability of having FA; respectively[OR = 1.110 (%95 CI = 1.055–1.168) *p* < 0.05]and [OR = 1.805 (%95 CI = 1.100–2.961) *p* < 0.05]; also increased significantly the probability of having NES, respectively; [OR = 4.073 (%95 CI = 2.334–7.109) *p* < 0.001] and [OR = 1.110 (%95 CI = 1.044–1.179) *p* < 0.001](Table 4). Similar to the results of this study, other studies showed that BMI level was an important predictor of FA (Mutlu and Mutlu 2022) and NES in adults (Ahmad et al. 2019). The emergence of these findings may be influenced by the inability of university students to cope with the stressors they encounter and the resulting development of unhealthy sleep patterns and eating behavior, leading to depression, obesity, and NES.

Increased stress levels in university students (Kuru Sonmez, Yakut, and Cankal 2022), high depressive symptoms (Yazici and Ak 2008), anxiety and anger (Alagoz et al. 2024), being male, staying in a student dormitory, circle of friends, alcohol consumption, and not consuming any vegetables or fruit increase the likelihood of smoking (Thomas, Caputi, and Wilson 2014). In a recent study, university students who smoked were found to be more likely to skip breakfast than non‐smokers (Khan et al. 2024), and the likelihood of e‐cigarette use increased in young people with eating disorders (binge eating syndrome and NES) (Smith et al. 2024). In another study, smoking was found to be an important predictor of food addiction (Abdelwanees et al. 2024). In a study conducted on adults, the rate of active smoking was 29.3% in those with night‐eating syndrome and 33.8% in individuals under psychological distress but that smoking had no effect on night‐eating syndrome and being under psychological distress (Sayed Ahmed et al. 2024). In this study, smoking was observed in more than 30.0% of the students with food addiction, night‐eating syndrome, and moderate–severe depression (Table 1), but it was determined that smoking was not a significant predictor of FA and NES (Table 4). Additionally, it was found that students with FA (30.7%) and NES (28.6%) had a high rate of receiving psychological treatment (Table 1). Yildiz et al. reported that night‐eating syndrome was observed more in university students who slept less than six hours, smoked, consumed more tea or coffee, and had a disease diagnosed by a physician (Yildiz et al. 2021). Studies on tobacco use and eating habits are limited, and more research is needed, especially on the psychological states and eating disorders of smokers.

In a study on the level of depression in university students, it was determined that the level of depression increased by 5% when the serum 25‐OH‐D levels decreased by 1 ng/mL, and the presence of depression was found to be the only independent factor associated with NES (Fallah et al. 2024). In a study conducted on students in Ecuador, no difference was observed between students' food addictions according to regional differences; however, it was reported that age, female gender, high psychological fluctuations, negative mood, impulsivity, and anxiety triggered food addiction (Reivan Ortiz et al. 2024). In this study, the increase in students' depression levels was a factor that significantly increased the likelihood of FA [OR = 2.344 (%95 CI = 1.633–3.364) *p* < 0.001] and NES [OR = 5.069 (%95 CI = 3.023–8.498) *p* < 0.001](Table 4), and it was observed that both FA and NES were at a higher rate in those with moderate–severe depression levels (Table 3). In addition, although receiving psychological support was not found to be directly effective in NES [OR = 1.571 (%95 CI = 0.892–2.770) *p* > 0.05], it posed a significant risk in terms of FA [OR = 2.125(%95 CI = 1.402–3.222) *p* < 0.001] (Table 4). Although the relationship between eating disorders and certain mental health problems, such as anxiety, depression, and substance use disorder, has been examined in the literature (Alagoz et al. 2024; Fallah et al. 2024; Hong et al. 2020), research revealing this relationship is limited. Therefore, further research is needed on the comparative analyses of various mental health problems and their relationships with eating disorders.

Although it is known that individuals' eating habits are associated with many factors, it has been reported that mood changes may be the cause of eating disorders (Howells, Dunn, and Carter 2024; Hudson et al. 2007), and the incidence of depression may vary depending on different food preferences (Du et al. 2023; Ljungberg, Bondza, and Lethin 2020; Selvaraj et al. 2022). Studies in the literature have shown that food addiction is associated with anxiety (Cheah and Chin 2024), depression (Sanlier, Türközü, and Toka 2016), and night eating (Arslan et al. 2024; Nolan and Geliebter 2016) in university students. In this study, the evaluation of the FA, NES, and depression levels of university students together made a significant contribution to the literature. Although the relationship between these three concepts is rarely seen in the literature, Taymur et al. (2019) reported that mean YFAS scores increased significantly as NEQ scores increased in adults. A study conducted by Nolan and Geliebteron on university students found that the mean YFAS score had a positive and statistically significant relationship with mean NEQ and SDS (Zung Self‐report Depression Scale) scores (Nolan and Geliebter 2016). In our study, a positive, statistically significant relationship was found between the mean YFAS score and the mean NEQ and BDI scores for the students in general and both male and female students separately, and it was determined that as the mean NEQ score increased, the mean BDI score increased, as well (Figure 2). Therefore, it is evident that these three variables, in conjunction with each other, significantly affect the lives of university students.

This study has some limitations. For example, the anthropometric measurements of the students were recorded based on self‐report. Also, the study sample was selected from a single center. Moreover, the study data were collected online.

## 5 Conclusions

Some reasons, such as changes in social environment, feeling of freedom, separation from family, and economic shortcomings, can lead to depression, FA, and NES in university students. Worsening of students' emotional states causes the prevalence of eating disorders, an increase in the risk for obesity and cardiovascular diseases, the inclusion of addictive bad habits such as smoking to life, and the adoption of unbalanced meal habits. Screening programs should be carried out particularly for university students at regular intervals throughout their education and risks should be identified. They should be followed by physicians, nurses, and dietitians and provided with education, consultancy, treatment, and healthy diet support, all of which will be useful in terms of raising social awareness. Therefore, conducting multi‐center studies with face‐to‐face interviews with risk groups with different sociodemographic characteristics will contribute to the field.

## Author Contributions

**Sinem EREM:** conceptualization (lead); data curation (lead); formal analysis (lead); investigation (lead); methodology (lead); writing – original draft (lead); writing – review and editing (lead). **Deniz EREN:** conceptualization (lead); data curation (lead); formal analysis (lead); investigation (lead); methodology (supporting); writing – original draft (lead); writing – review and editing (lead).

## Conflicts of Interest

The authors declare no conflicts of interest.

## Data Availability

The datasets generated and/or analyzed throughout this study are publicly unavailable.
